# Validation of 30-Day Pediatric Hospital Readmission Risk Prediction Models

**DOI:** 10.1001/jamanetworkopen.2024.59684

**Published:** 2025-02-13

**Authors:** Alison R. Carroll, Matthew Hall, Mitch Harris, Michael S. Carroll, Katherine A. Auger, Matthew M. Davis, Denise M. Goodman, Derek J. Williams

**Affiliations:** 1 Division of Pediatric Hospital Medicine, Department of Pediatrics, Vanderbilt University School of Medicine, Nashville, Tennessee; 2 Monroe Carell Jr. Children’s Hospital at Vanderbilt, Nashville, Tennessee; 3 Children’s Hospital Association, Lenexa, Kansas; 4 Quantitative Science Pillar, Stanley Manne Children’s Research Institute of Chicago, Chicago, Illinois; 5 Division of Hospital Medicine, Department of Pediatrics, Cincinnati Children’s Hospital Medical Center, Cincinnati, Ohio; 6 Nemours Children’s Health, Wilmington, Delaware; 7 Division of Critical Care Medicine, Ann & Robert H. Lurie Children’s Hospital of Chicago, Chicago, Illinois

## Abstract

**Importance:**

Accurate identification of hospital readmission risk during a current hospitalization may enhance decision-making, facilitate targeted systems-level interventions, and avoid preventable readmissions.

**Objective:**

To temporally and externally validate a suite of readmission risk prediction models across 48 children’s hospitals to assess their generalizability and feasibility for future clinical implementation.

**Design, Setting, and Participants:**

This prognostic study analyzed data from the Pediatric Health Information System (PHIS) database, which contains billing and resource use data from 48 US children’s hospitals, including the derivation hospital (DH) and 47 hospitals participating in the PHIS database (hereafter other PHIS hospitals). Children aged 18 years or younger discharged from these hospitals between January 1, 2016, to December 31, 2019, were included. This cohort was divided as specified into the 3 prediction models at the DH: 6 months or older with no recent hospitalizations (new admission model [NAM]), 6 months or older with 1 or more prior hospitalizations within the last 6 months (recent admission model [RAM]), and 6 months or younger (young infant model [YIM]). Data were analyzed from August 9 to December 1, 2023.

**Main Outcomes and Measures:**

The primary validation outcome was hospital-level discrimination measured with area under the receiver operating characteristic curve (AUROC). Predictors included demographic, clinical, and utilization variables. All-cause 30-day readmission was modeled for each hospital using logistic regression and parameter estimates from the DH. Calibration plots examined observed vs predicted outcome frequencies for each hospital.

**Results:**

In external validation, a total of 851 499 children were discharged from 48 hospitals (16 330 DH discharges and 835 169 other PHIS hospital discharges). The largest group of children was aged 5 to 14 years (281 193 [33.0%]). In temporal validation, the DH PHIS 2016-2018 cohort included 45 682 discharges. All-cause 30-day readmission rates were 7.2% for NAM, 35.5% for RAM, and 11.7% for YIM. The 2019 DH PHIS cohort included 16 330 discharges. All cause 30-day readmision rates were 7.2% for NAM, 35.1% for RAM, and 11.1% for YIM. Temporal validation demonstrated reduced discrimination across all 3 models (median AUROC, 0.65 [95% CI 0.62-0.67] for the NAM; 0.73 [95% CI 0.72-0.75) for RAM; 0.67 [95% CI 0.63-0.70) for the YIM compared with the original estimates (median AUROC 0.76 [95% CI 0.85-0.78] for the NAM; 0.84 [95% CI 0.83-0.84] for the RAM; 0.79 [95% CI 0.77-0.80] for the YIM). Overall readmission rates were 5.9% for NAM, 30.1% for RAM, and 7.6% for YIM. External validation yielded similiar findings as the temporal validation, although with demonstrable variation in performance across hospitals (median [range] AUROC, 0.64 [0.60-0.68] for the NAM; 0.73 [0.64-0.80] for the RAM; 0.65 [0.53-0.74] for the YIM). Most hospitals were poorly calibrated, with both significant overestimation and underestimation of observed risk. Of 47 other PHIS hospitals, only 3 for the RAM (6.4%) and 9 for both the NAM and YIM (19.1%) were adequately calibrated.

**Conclusions and Relevance:**

This prognostic study found that the readmission risk prediction models had reduced predictive accuracy across time and variability in hospital-level performance. These findings stress the importance of local validation prior to clinical implementation and suggest opportunities to improve generalizability, including multicenter derivation and expansion of candidate predictors.

## Key Points

**Question:**

How do single center–derived models for predicting all-cause 30-day readmission risk perform within a large sample of US children’s hospitals?

**Findings:**

In this prognostic study involving 851 499 children discharged from 48 children’s hospitals, the models’ predictive performance declined in both temporal validation at the derivation hospital and external validation across 47 children’s hospitals. The models retained discriminatory power, but there was variability in hospital-level performance.

**Meaning:**

Single center–derived readmission risk models showed reduced predictive accuracy across time and variability in hospital-level performance, suggesting the need for more accurate and generalizable models and rigorously designed impact analyses to ascertain clinical utility in reducing preventable use and improving outcomes.

## Teaser

This prognostic study assesses the multihospital-level accuracy and variability of readmission risk prediction models.

## Introduction

If readmission were a diagnosis, it would outrank all other hospital diagnoses for children outside of the newborn period in the US.^1^ As of 2020, pediatric 30-day readmission rates were 11.4%, with costs nearly 1.4 times the cost of an index admission, a ratio higher than for all adult age groups.^2^ As such, much attention is paid to readmission rates by health systems and payers.^3,4,5,6,7,8,9^ Readmissions are also burdensome and disruptive for patients and families. Considerable efforts have focused on reducing readmissions, including a variety of hospital-to-home transition interventions.^10^ While 1 large randomized clinical trial found that nurse home visits did not prevent readmissions, other studies targeting higher-risk populations reported significantly reduced emergency department (ED) revisits and readmissions.^10,11,12,13,14,15^

Solutions that harness real-time health system data (ie, clinical, demographic, and socioeconomic data in the electronic health record [EHR]) to objectively define readmission risk may be a factor in improved effectiveness of hospital-to-home transitions by identifying high-risk patients early in a hospitalization. Challenges with existing readmission prediction models include being limited in scope (ie, focus on a single diagnosis),^16^ being proprietary,^17,18^ having poor discriminative ability,^19^ or lacking validation.^18,20^ Multicenter-derived readmission models have also included administrative data not routinely available at the point of care,^8,21,22,23^ limiting their utility for informing clinician decision-making during a current admission.

Goodman et al^20^ used EHR data from 48 019 hospitalizations at a single, freestanding children’s hospital (ie, derivation hospital [DH]) to predict all-cause 30-day readmission across 3 cohorts: (1) children age 6 months or older with no prior hospitalizations in the last 6 months (new admission model [NAM]), (2) children age 6 months or older with 1 or more hospitalizations within 6 months (recent admission model [RAM]), and (3) children age younger than 6 months (young infant model [YIM]). The resulting readmission risk prediction models included 4 to 7 predictors, and each demonstrated good to excellent discrimination as measured by area under the receiver operating characteristic curve (AUROC) ranging from 0.76 to 0.83 (eTable 1 in Supplement 1). These models are novel because they were designed to be integrated into the EHR and used at the point of care to inform disposition and hospital-to-home transition decisions. The generalizability of these models to other settings is unknown. Rigorous model validation requires evaluating the model’s performance using the originally specified parameters in a new population or setting.^24^ This often overlooked step is critical to avoid promulgation of badly performing models that do not help, or worse, are harmful.^25^ However, translating these off-the-shelf models, when validated, to other hospitals could help overcome common time, expertise, and resource barriers to facilitate more widespread adoption. Therefore, we sought to temporally and externally validate this suite of readmission risk prediction models across 48 US children’s hospitals to assess their generalizability and feasibility for future clinical implementation.^24^

## Methods

### Design, Data Source, and Population

We sourced data from the Children’s Hospital Association Pediatric Health Information System (PHIS) administrative database, which contains billing and resource use data from 48 US children’s hospitals, including the DH, across 27 states and 17 of the 20 largest metropolitan areas. To distinguish the DH from the remaining 47 hospitals in the PHIS database, we refer to them as *other PHIS hospitals*. All children aged 18 years or younger hospitalized (inpatient or observation status) at the DH or other PHIS hospitals between January 1, 2016, and December 31, 2019, were considered for inclusion. We excluded encounters resulting in death or transfer to another facility. The Vanderbilt University Medical Center Institutional Review Board deemed this prognostic study exempt from ethics review and informed consent requirement because it was not human participant research. We adhered to the Transparent Reporting of a Multivariable Prediction Model for Individual Prognosis or Diagnosis (TRIPOD) reporting guideline.^26^

### Predictor Variables

Candidate predictors for the original models^20^ were chosen according to literature review^18,27,28,29,30^ and clinical judgment. Final predictors were selected using bivariate significance testing for parsimony (Goodman et al^20^ provides a full methodological explanation). The final predictor set included the following EHR-derived demographic and clinical characteristics: age at admission, race and ethnicity (Hispanic, non-Hispanic Black, non-Hispanic White, and non-Hispanic other [including American Indian or Alaska Native, Asian, Native Hawaiian or Other Pacific Islander, or multiracial] or unknown/unspecified), and insurance type (government, commercial, and other [including uninsured, self-insured, and multiple insurances] or unspecified); as well as medical history, including prior acute care use (hospital and ED discharges) and select prior procedures (invasive or noninvasive ventilation, central venous catheter placement, or transfusion) within 6 months of index admission; and measures of acute care use for the index admission, such as principal diagnosis (10 common diagnosis groups and 1 other), length of stay (LOS), admission location (ED vs other), service line (medical vs surgical), and use of intensive care. Race and ethnicity data were collected because they were included as predictor variables in the original models.

Modification for the service line variable, which was unavailable in the PHIS database, was required. Instead, surgical encounters were identified as those with operating room charges or with any *International Statistical Classification of Diseases and Related Health Problems, Tenth Revision* operating room procedure code.^31^ All other encounters were considered medical encounters. eTable 1 in Supplement 1 provides the model cohort definition, predictors, and predictive performance. Approximately 6% of the data had missing race and ethnicity information, and this missing variable was categorized as other. No hospitals were excluded due to data integrity issues.

### Readmission Outcome

The models were designed to predict all-cause 30-day readmission, defined as any hospitalization (observation or inpatient) occurring within 30 calendar days of the index discharge to the same hospital, including both planned (ie, elective) and unplanned readmissions. Each hospitalization was treated as an index encounter. Thus, it was possible for readmission encounters to also serve as a new index encounter.

### Validation Steps

**Figure 1.**
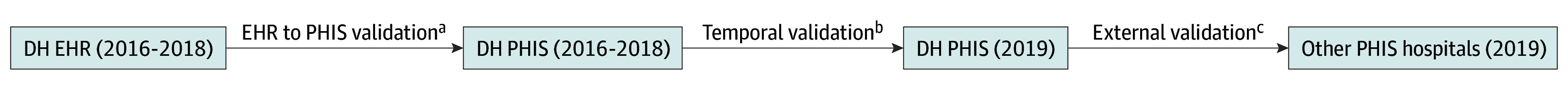
Steps for Statistical Analysis AUROCs for electronic health record (EHR) to Pediatric Health Information System (PHIS) validation were 0.76 (95% CI, 0.75-0.77) for new admission model, 0.83 (95% CI, 0.82-0.84) for recent admission model, and 0.80 (95% CI, 0.79-0.82) for young infant model. DH indicates derivation hospital. ^a^AUROCs from Goodman et al.^20^ ^b^Temporal validation AUROCs are provided in eTable 2 in Supplement 1. ^c^External validation AUROCs are provided in Table 2.

As the original models^20^ were developed using EHR data at a single, tertiary care children’s hospital, we first validated the model construct (hereafter DH PHIS) by fitting the DH models as originally specified to the 2016 to 2018 PHIS data (the derivation study period) for the DH. Index admission dates between January 1, 2016, and December 31, 2018, were included, and 30-day follow-up required following some cases into 2019 to measure 30-day readmission. We anticipated that discrimination (ie, AUROC) should be essentially identical (Figure 1, EHR to PHIS validation).

We then proceeded with temporal validation, fitting the DH PHIS models estimated using the 2016 to 2018 data to the 2019 DH PHIS data (Figure 1, temporal validation). Index admission dates were January 1 to December 31, 2019, and 30-day follow-up required following some cases into 2020 to measure 30-day readmission. Given the substantial changes in use during the COVID-19 pandemic, we did not include index encounters after 2019.^32^

For external validation, we fit the DH PHIS models to the 2019 data for other PHIS hospitals (Figure 1, external validation). Characteristics of each population were summarized as counts and percentages for categorical variables and as medians and IQRs for continuous variables.

### Statistical Analysis

For each analysis, logistic regression models were fit using the same modeling procedures as described for the DH cohorts, substituting the parameter estimates specified by the DH PHIS models. Patient was used as a random effect, as was done in the original models. Predictive performance for each model was evaluated using discrimination and calibration. Discrimination quantifies each model’s ability to distinguish between encounters with and encounters without all-cause 30-day readmission, and it was measured in this study using the AUROC, wherein a value of 1 indicates perfect discrimination and 0.5 is equivalent to chance (ie, coin flip). In general, an AUROC of 0.7 to 0.8 is considered acceptable, 0.8 to 0.9 is considered excellent, and more than 0.9 is considered outstanding.^33,34^ Median AUROCs with 95% CIs were estimated across the other PHIS hospitals to summarize the aggregate performance of the DH PHIS models.

Calibration measures the degree of agreement between predicted and observed outcome frequencies across the full range of predicted risk.^35^ We created calibration plots of predicted vs observed risk for all-cause 30-day readmission for each PHIS hospital, evaluating for significant differences in calibration slope and intercept (null hypotheses of intercept = 0; slope = 1).

All analyses were performed from August 9 to December 1, 2023, using SAS, version 9.4 (SAS Institute Inc). Two-sided *P* < .05 was considered statistically significant.

## Results

In external validation, a total of 851 499 children were discharged from 48 children’s hospitals. In 2019, there were 16 330 discharges from the DH and 835 169 discharges from the other PHIS hospitals (Table 1). The largest group of children was aged 5 to 14 years (281 193 [33.0%]). There were 165 020 (19.3%) Hispanic children, 409 419 (48.0%) non-Hispanic Black children, and 165 364 (19.4%) non-Hispanic White children.

**Table 1.** Patient Characteristics in the DH vs Other PHIS Hospitals, 2019

Characteristic	Patients, No. (%)		Other PHIS hospitals, median (IQR), %^a^
	DH	Other PHIS hospitals	
No. of discharges	16 330	835 169	16 111 (13 056-21 449)
Age at admission			
<2 mo	1430 (8.8)	145 075 (17.4)	13.7 (8.8-21.3)
2-5 mo	1049 (6.4)	49 967 (6.0)	5.9 (5.2-6.8)
6-11 mo	1186 (7.3)	49 790 (6.0)	6.0 (5.4-6.7)
1-4 y	4831 (29.6)	200 740 (24.0)	25.1 (21.7-26.7)
5-14 y	5525 (33.8)	275 668 (33.0)	33.2 (29.5-37.1)
≥15 y	2309 (14.1)	113 929 (13.6)	13.7 (11.6-15.1)
ED admittance	9009 (55.2)	451 312 (54.0)	55.5 (45.8-62.3)
Insurance type			
Government	8820 (54.0)	449 385 (53.8)	54.0 (48.7-61.0)
Commercial	7312 (44.8)	335 211 (40.1)	38.3 (32.9-45.7)
Other or unspecified^b^	198 (1.2)	50 573 (6.1)	4.9 (2.7-7.3)
LOS, d			
<3	10 487 (64.2)	600 612 (71.9)	72.3 (68.2-74.6)
≥3	5843 (35.8)	234 557 (28.1)	27.7 (25.4-31.8)
PICU admission	3495 (21.4)	112 161 (13.4)	11.7 (10.0-16.4)
Primary diagnosis category, age <6 mo			
Other^c^	1174 (47.4)	130 547 (66.9)	53.5 (48.6-71.4)
ALTE or BRUE	60 (2.4)	2085 (1.1)	1.0 (0.6-1.7)
Cardiac	171 (6.9)	6503 (3.3)	3.7 (2.1-5.1)
Congenital anomalies	199 (8.0)	7610 (3.9)	4.2 (2.1-7.9)
Esophageal reflux	37 (1.5)	2650 (1.4)	1.5 (0.9-2.1)
Fever	85 (3.4)	3285 (1.7)	1.9 (1.4-2.5)
Respiratory, lower (bronchiolitis and pneumonia)	507 (20.5)	27 637 (14.2)	17.8 (10.7-21.6)
Respiratory, upper	52 (2.1)	2555 (1.3)	1.6 (0.9-2.1)
Neonatal jaundice	84 (3.4)	7080 (3.6)	3.6 (2.4-5.0)
Pyloric stenosis	42 (1.7)	2393 (1.2)	1.5 (0.8-1.8)
UTI and pyelonephritis	68 (2.7)	2697 (1.4)	1.3 (0.8-2.4)
Primary diagnosis category, age ≥6 mo			
Other^c^	8931 (64.5)	444 112 (69.4)	69.0 (64.7-72.5)
Appendicitis	69 (0.5)	12 919 (2.0)	1.8 (1.2-2.6)
Asthma	938 (6.8)	29 814 (4.7)	4.8 (3.1-6.3)
CNS shunt	84 (0.6)	2650 (0.4)	0.4 (0.3-0.5)
Dehydration or GI infection	569 (4.1)	19 753 (3.1)	2.8 (2.3-3.7)
Fever	128 (0.9)	3289 (0.5)	0.5 (0.3-0.7)
Respiratory, lower (bronchiolitis)	489 (3.5)	24 694 (3.9)	3.8 (2.4-5.1)
Respiratory, lower (pneumonia)	1483 (10.7)	45 352 (7.1)	6.7 (5.5-8.4)
Respiratory, upper	304 (2.2)	10 220 (1.6)	1.5 (1.2-2.0)
Seizure	663 (4.8)	38 736 (6.1)	5.7 (4.5-7.2)
Sickle cell anemia	193 (1.4)	8588 (1.3)	1.0 (0.6-1.8)
Any prior select procedures within 6 mo before admission	5387 (33.0)	196 507 (23.5)	23.4 (18.7-27.3)
Prior use within 6 mo before admission	6448 (39.5)	289 950 (34.7)	35.2 (31.7-38.9)
Race and ethnicity^d^			
Hispanic, any race	5518 (33.8)	159 502 (19.1)	13.2 (3.8-31.6)
Non-Hispanic Black	6027 (36.9)	403 392 (48.3)	48.4 (33.6-64.1)
Non-Hispanic White	2849 (17.4)	162 515 (19.5)	17.5 (10.5-23.8)
Non-Hispanic other or unknown/unspecified^e^	1936 (11.9)	109 760 (13.1)	10.0 (7.2-16.9)
Service line			
Medical	13 804 (84.5)	683 821 (81.9)	85.5 (77.5-88.3)
Surgical	2526 (15.5)	151 348 (18.1)	14.5 (11.7-22.5)

Abbreviations: ALTE, apparent life-threatening event; BRUE, brief, resolved, unexplained event; CNS, central nervous system; DH, derivation hospital; ED, emergency department; GI, gastrointestinal; LOS, length of stay; PHIS, Pediatric Health Information System; PICU, pediatric intensive care unit; UTI, urinary tract infection.

^a^ Median percentage for each characteristic at the other PHIS hospitals excluding DH.

^b^ Other insurance type included uninsured, self-insured, and multiple insurances.

^c^ Any other diagnosis category not otherwise listed.

^d^ Race and ethnicity were derived from the PHIS database.

^e^ Other race and ethnicity included American Indian or Alaska Native, Asian, Native Hawaiian or Other Pacific Islander, and multiracial.

### EHR to PHIS Validation

The 2016 to 2018 DH cohort included 45 682 discharges. All-cause 30-day readmission rates for the model subpopulations were 7.2% (1855 of 25 750 discharges) for the NAM, 35.5% (4539 of 12 794 discharges) for the RAM, and 11.7% (831 of 7128 discharges) for the YIM (eTable 2 in Supplement 1). As expected, the predictive performance of the DH PHIS models was essentially identical to the previously published values for the DH cohort (Figure 1). Specifically, the AUROC for the NAM was 0.76 (95% CI, 0.75-0.78) in the DH PHIS models compared with 0.76 (95% CI, 0.75-0.77) in the original DH cohort, AUROC for the RAM was 0.84 (95% CI, 0.83-0.84) compared with 0.83 (95% CI, 0.82-0.84), and AUROC for the YIM was 0.79 (95% CI, 0.77-0.80) compared with 0.80 (95% CI, 0.79-0.82).^20^

### Temporal Validation

The 2019 DH cohort included 16 330 discharges. All-cause 30-day readmission rates were 7.2% (676 of 9363 discharges) for the NAM, 35.1% (1574 of 4488 discharges) for the RAM, and 11.1% (275 of 2479 discharges) for the YIM. The AUROC was 0.65 (95% CI, 0.62-0.67) for the NAM, 0.73 (95% CI, 0.72-0.75) for the RAM, and 0.67 (95% CI, 0.63-0.70) for the YIM (Figure 1, temporal validation; eTable 2 in Supplement 1). These AUROCs appeared lower than the 2016 to 2018 estimates.

### External Validation

The 2019 other PHIS hospitals cohort included 835 169 discharges, with median (range) hospital-level discharges of 16 111 (13 056-21 449). The readmission rates were 5.9% (27 798 of 470 202 discharges) for the NAM, 30.1% (51 177 of 169 925 discharges) for the RAM, and 7.6% (14 842 of 195 042 discharges) for the YIM (Table 2). Overall, 2.7% of patients had more than 1 readmission. Table 1 compares the characteristics of 2019 DH vs other PHIS hospital encounters and demonstrates differences in age at admission, race and ethnicity, LOS, and frequency of pediatric intensive care unit admission, prior select procedures, and prior use.

**Table 2.** External Validation: Primary Outcome and Discrimination for the DH PHIS and Other PHIS Hospitals, 2019

	DH PHIS (n = 16 330)			Other PHIS hospitals (n = 835 169)		
	NAM	RAM	YIM	NAM	RAM	YIM
No. of discharges	9363	4488	2479	470 202	169 925	195 042
30-d Readmission, No. (%)	676 (7.2)	1574 (35.1)	275 (11.1)	27 798 (5.9)	51 177 (30.1)	14 842 (7.6)
Other PHIS hospital–level median readmission (IQR), %	NA	NA	NA	5.9 (5.4-6.3)	29.7 (27.4-33.0)	8.4 (6.5-9.9)
DH-level AUROC (95% CI)	0.65 (0.62-0.67)	0.73 (0.72-0.75)	0.67 (0.63-0.70)	NA	NA	NA
Other PHIS hospital–level median AUROC (range)	NA	NA	NA	0.64 (0.60-0.68)	0.73 (0.64-0.80)	0.65 (0.53-0.74)

Abbreviations: AUROC, area under the receiver operating characteristic curve; DH, derivation hospital; NA, not applicable; NAM, new admission model; PHIS, Pediatric Health Information System; RAM, recent admission model; YIM, young infant model.

**Figure 2.**
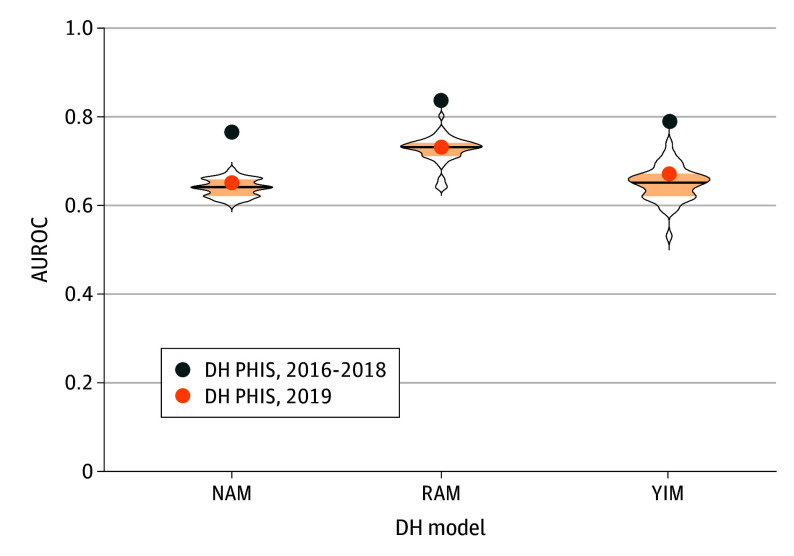
Violin Plot of Hospital-Level Median Area Under the Receiver Operating Characteristic Curves (AUROCs) of the External Validation of the 2019 Other Pediatric Health Information System (PHIS) Hospitals Cohort The horizontal line in the middle of each plot represents the hospital-level median AUROC for each derivation hospital (DH) model. The shaded area above and below the median represents the 25th and 75th IQR, respectively. The width of each plot represents the density of observations across all other PHIS hospitals. NAM indicates new admission model; RAM, recent admission model; and YIM, young infant model.

Median AUROC values for the 2019 other PHIS hospitals were similar to those estimated for the 2019 DH cohort (Table 2 and Figure 1, external validation). However, variation in median (range) AUROCs was observed across individual hospitals (Figure 2), with 0.64 (0.60-0.68) for the NAM, 0.73 (0.64-0.80) for the RAM, and 0.65 (0.53-0.74) for the YIM. To explore the possible reasons for hospital-level variability in model performance, we used the RAM to compare hospital-level characteristics across tertiles of AUROC values for the other PHIS hospitals (eTable 3 in Supplement 1). We noted differences in median (IQR) readmission rate between the lowest and highest tertiles (28.3% [26.4%-29.3%] vs 32.9% [28.4%-35.4%]). There was a higher percentage of patients with 4 or more prior uses in the highest vs lowest tertile (33.8% vs 28.4%) and patients with 1 or more prior select procedures (49.9% vs 45.9%).

### Calibration

**Figure 3.**
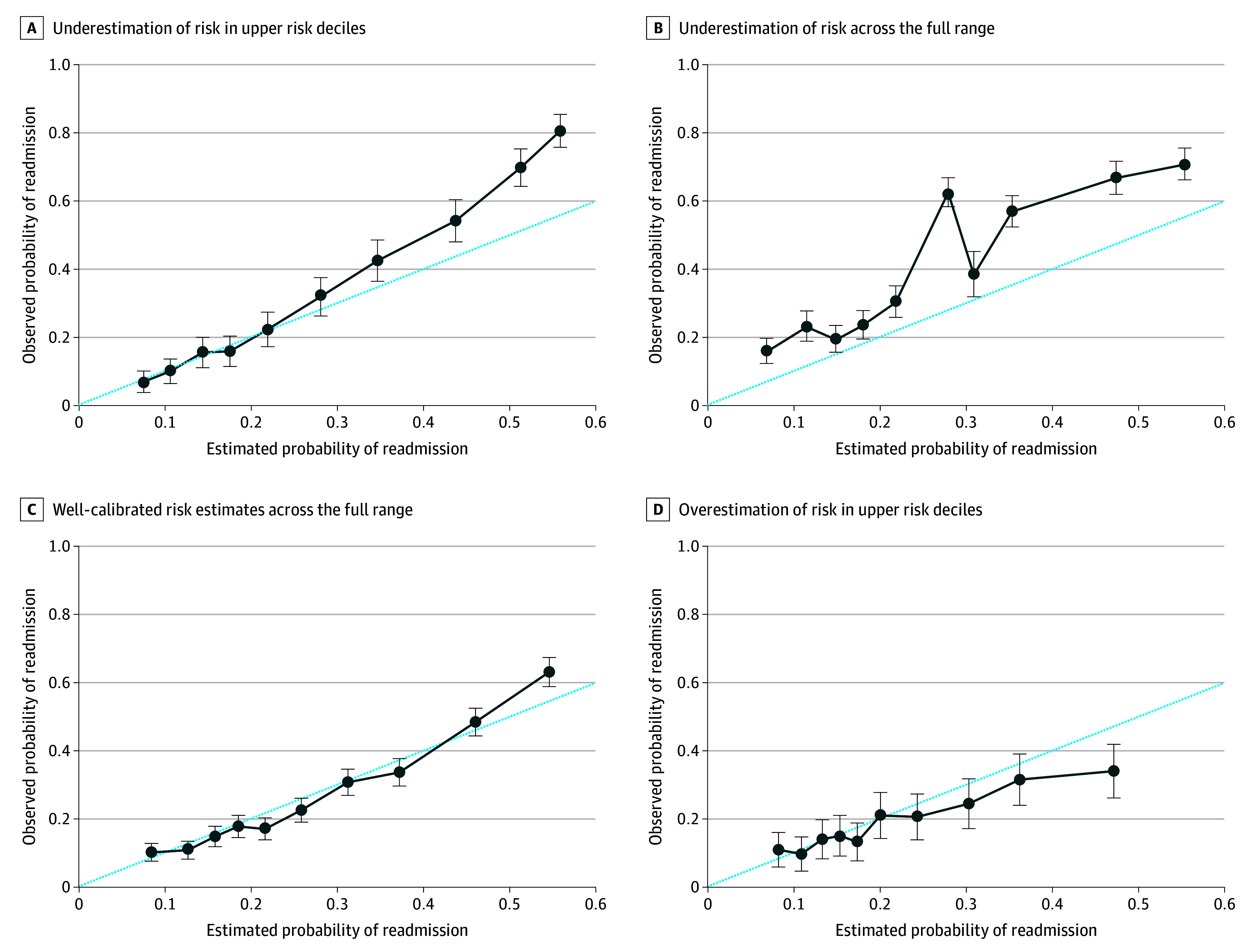
Four Calibration Plots for the Recent Admission Model (RAM) of the 2019 Other Pediatric Health Information System Hospitals Ideal calibration is represented by the 45-degree dotted line through the origin. The circles represent observed risk, with whiskers representing 95% CIs at each decile of predicted risk.

Calibration plots were generated for each of the PHIS hospitals to compare agreement between observed and predicted risk probabilities. Of the 47 other PHIS hospitals, only 3 for the RAM (6.4%) and 9 for both the NAM and the YIM (19.1%) were adequately calibrated. Figure 3 shows calibration plots for the RAM cohort at 4 hospitals to demonstrate variation in calibration performance, including risk underestimation as observed risk increased (Figure 3A), underestimation across the full range (Figure 3B), well-calibrated estimates (Figure 3C), and risk overestimation as observed risk increased (Figure 3D).

## Discussion

Using a multicenter cohort of children discharged from 48 US children’s hospitals, we evaluated the performance of a suite of single center–derived models for predicting all-cause 30-day readmissions. Temporal validation of the original readmission risk prediction models^20^ within the DH yielded reduced discriminatory performance (Figure 1, temporal validation). When the models were applied externally across 47 other children’s hospitals in the PHIS database, model discrimination varied at individual hospitals, although aggregate performance approximated the results from the temporal validation (Figure 1, external validation). The RAM demonstrated the best discrimination (median [range] AUROC, 0.73 [0.64-0.80]), which is on par or better than other published pediatric^16,18,19,21,36,37^ and adult^27,28,38,39^ readmission models.

Degradation in predictive performance is expected when models are applied in new settings,^40^ and several possibilities may explain the noted declines in this study. In the temporal DH validation (presumably stable population-level risk), seasonal differences in circulating respiratory viruses and other common infectious pathogens, including variation in risk for severe disease outcomes, may be factors in the risk for reuse that were not well captured in the models. Other population-level differences may further play a role in drift in discriminatory power. The DH models also included just a few predictors and imposed categorical variable transformations for parsimony and ease of interpretation, decisions that may be associated with information loss, especially if predictors are omitted or oversimplified.

These same factors may be associated with reduced discrimination in external settings. For example, the prevalence of all-cause 30-day readmission varied between the DH and each of the other PHIS hospitals. Median predictive performance across the other PHIS hospitals was overall consistent with performance under previously published pediatric risk prediction models^23,41^ but was suboptimal, with AUROCs ranging from 0.65 to 0.73.^33^ Performance was best for the RAM (AUROC, 0.73) likely because prior use, a requirement for RAM cohort inclusion, was one of the strongest predictors of subsequent use. Supporting this assertion was that the RAM cohort had the highest prevalence of children with medical complexity, a population at high risk for recurrent use.^42^ The YIM had the widest variability in model performance across hospitals and low overall risk for readmission. In addition, only 4 predictors were included in the YIM, suggesting that unmeasured heterogeneity in young infant care was associated with the observed variation. Considering new predictors to better capture important variation within this population would likely play a role in improved performance. Regardless of the reasons, the observed temporal and hospital-level variability suggests each model could potentially be improved and highlights the importance of local validation prior to clinical implementation.

Two studies have applied the LACE Index (LOS, acuity of admission, comorbidities, and number of ED visits in the 6 months before admission), an adult-derived 30-day readmission model, to pediatric data.^29,43^ In both studies, discrimination (AUROCs of 0.68 [single center] and 0.70 [multicenter]) was similar to discrimination in the DH PHIS models in the current study.^18,44^ However, there may be limited clinical utility of the LACE Index in pediatric cohorts given its reliance on an adult-specific comorbidity measure (ie, Charlson Comorbidity Index) and that it is calibrated to use variables collected at discharge. Additionally, efforts to adapt the LACE Index by incorporating an expanded set of demographic variables have yielded suboptimal results in children.^45^ The DH PHIS models are specific to pediatric cohorts, suggesting they may be a preferable starting point over adult-derived models.

While the DH models overcame several limitations noted in prior studies, important opportunities to improve performance remain. Multicenter derivation of new models could increase generalizability and discriminatory power. Advanced modeling approaches, such as maintaining continuous predictors vs dichotomizing variables, leveraging data transformation (eg, spline functions) to relax linearity assumptions, and modeling higher-order interactions, are techniques that help to avoid information loss. The inclusion of additional predictors, such as the presence and degree of medical complexity, illness severity measures, and health-related social needs, may play a role in enhanced performance. For example, medical complexity, and comorbidities more generally,^36,37,46^ are key risk factors for readmission, but these constructs were not well captured in the original models. While these factors can be difficult to define using EHR data from an index encounter, querying prior encounters (ie, 1-year lookback) could be conducted to classify comorbidities using validated diagnostic classification systems, such as the pediatric Complex Chronic Conditions classification system^47^ or the Pediatric Medical Complexity Algorithm.^48^ Similarly, reporting area- and patient-level measures of social needs and assets is increasingly common within EHRs, and these data may help predict reuse unexplained by baseline health status or illness acuity. Large language models that mine notes and other unstructured EHR fields could also be factors in enhanced performance.^49^ The predictors in the DH models may serve as a useful starting point for hospitals wanting to use risk models to guide resource allocation and target hospital-to-home transition interventions where they are most needed. However, differences in model performance highlight the need for local validation, recalibration, and potentially model updating prior to clinical adoption.

### Limitations

This study has several limitations. First, we were unable to ascertain readmissions to hospitals not participating in the PHIS database, resulting in potential misclassification and underreporting of readmissions. However, prior studies have demonstrated that different hospital readmissions are less common within children’s hospitals than general hospitals.^50^ We did not include general hospitals that provide care to children, and the models may perform differently in those settings. Second, patterns of hospital use changed after the onset of the COVID-19 pandemic. Future studies should include contemporary data when designing, validating, or updating new models. Third, the original models^20^ did not differentiate between planned and unplanned readmissions. No differentiation may affect generalizability for hospitals that have substantially more or fewer planned readmissions.

## Conclusions

In this prognostic study, temporal validation of a suite of readmission risk prediction models at the same DH and external validation at 47 other children’s hospitals demonstrated reduced discriminatory performance compared with performance in the original derivation study. Hospital-level variability in predictive performance suggests the need to create more accurate and generalizable models as well as the need for rigorously designed impact analyses to ascertain clinical utility in reducing preventable use and improving outcomes.

## Notes

Supplement 1. **eTable 1.** Model Cohort Definition, Predictors and Predictive Performance of Original Goodman et al. Study **eTable 2.** Temporal Validation: Readmission Prevalence and Performance of Prognostic Models Comparing Derivation Hospital (DH) PHIS Models, 2016-2018 to DH PHIS, 2019 **eTable 3.** Patient-Level Encounter Characteristics for RAM Cohort by AUROC Tertile

Supplement 2.
Data Sharing Statement
