# Supplementary material for: Validation of 30-Day Pediatric Hospital Readmission Risk Prediction Models
Source: JAMA Netw Open. 2025 Feb 13;8(2):e2459684. doi: 10.1001/jamanetworkopen.2024.59684 (PMC11826366; doi:10.1001/jamanetworkopen.2024.59684)
Supplement: Supplement 1. — eTable 1. Model Cohort Definition, Predictors and Predictive Performance of Original Goodman et al. Study eTable 2. Temporal Validation: Readmission Prevalence and Performance of Prognostic Models Comparing Derivation Hospital (DH) PHIS Models, 2016-2018 to DH PHIS, 2019 eTable 3. Patient-Level Encounter Characteristics for RAM Cohort by AUROC Tertile [file jamanetwopen-e2459684-s001.pdf]

Supplemental Online Content

Carroll AR, Hall M, Harris M, et al. Validation of 30-day pediatric hospital readmission risk prediction models. *JAMA Netw Open*. 2025;8(2):e2459684. doi:10.1001/jamanetworkopen.2024.59684

eTable 1. Model Cohort Definition, Predictors and Predictive Performance of Original Goodman et al. Study

eTable 2. Temporal Validation: Readmission Prevalence and Performance of Prognostic Models Comparing Derivation Hospital (DH) PHIS Models, 2016-2018 to DH PHIS, 2019

eTable 3. Patient-Level Encounter Characteristics for RAM Cohort by AUROC Tertile

This supplemental material has been provided by the authors to give readers additional information about their work.

© 2025 Carroll AR et al. *JAMA Network Open*

eTable 1. Model Cohort Definition, Predictors and Predictive Performance of Original Goodman et al. Study

**Model Name and Cohort Definition**	**Predictors**	**AUROC[a] (95% CI)**
**Young Infant Model (YIM)**<br>Children <6 mos	Length of stay; Primary diagnosis category (10 common categories and "other" using ICD-10 codes); Prior procedure in 6 mos before admission; Prior utilization in 6 mos before admission (4 predictors)	80.3 (78.8-81.9)
**New Admission Model (NAM)**<br>Children ≥6 mos with no prior hospitalizations within last 6 mos	YIM predictors + ED admittance (Y/N); Race and ethnicity (Hispanic, Non-Hispanic Black, Non-Hispanic White, Non-Hispanic other or unknown/unspecified) (6 predictors)	76.1 (75.0-77.2)
**Recent Admission Model (RAM)**<br>Children ≥6 mos with 1+ prior hospitalizations within last 6 mos	YIM predictors + ED admittance (Y/N); Insurance (commercial, government or other); Service category (surgical vs. medical) (7 predictors)	83.1 (82.4-83.8)

[a]AUROC=area under the receiver operator curve

© 2025 Carroll AR et al. *JAMA Network Open*

eTable 2. Temporal Validation: Readmission Prevalence and Performance of Prognostic Models Comparing Derivation Hospital (DH) PHIS Models, 2016-2018 to DH PHIS, 2019

	DH PHIS 2016-2018 (n=45 682)			DH PHIS 2019 (n=16 330)		
	**NAM**	**RAM**	**YIM**	**NAM**	**RAM**	**YIM**
N Discharges	25 760	12 794	7128	9363	4488	2479
N Readmitted	1855	4539	831	676	1574	275
% Readmitted	7.2%	35.5%	11.7%	7.2%	35.1%	11.1%
AUROC (95% CI)	0.76 (0.75, 0.78)	0.84 (0.83, 0.84)	0.79 (0.77, 0.80)	0.65 (0.62, 0.67)	0.73 (0.72, 0.75)	0.67 (0.63, 0.70)

© 2025 Carroll AR et al. *JAMA Network Open*

eTable 3. Patient-Level Encounter Characteristics for RAM[a] Cohort by AUROC[b] Tertile

Characteristic	Overall	RAM AUROC 0.63-0.70	RAM AUROC 0.70-0.74	RAM AUROC 0.74-0.80	*P* value
No. Hospitals	48	12	24	12	
No. Discharges	174 413	55 691	84 996	33 726	
Median Readmission Rate %, [IQR]	29.7 [27.4, 33.1]	28.3 [26.4, 29.3]	29.9 [27.5, 33.1]	32.9 [28.4, 35.4]	<.001
ED admittance					
No	81 733 (46.9)	26 705 (48)	39 386 (46.3)	15 642 (46.4)	<.001
Yes	92 680 (53.1)	28 986 (52)	45 610 (53.7)	18 084 (53.6)	
Insurance					
Government	100 731 (57.8)	31096 (55.8)	48568 (57.1)	21067 (62.5)	<.001
Commercial	64 190 (36.8)	20141 (36.2)	32670 (38.4)	11379 (33.7)	
Other or unspecified	9492 (5.4)	4454 (8)	3758 (4.4)	1280 (3.8)	
Length of stay, d					
<3	106 861 (61.3)	34796 (62.5)	51733 (60.9)	20332 (60.3)	<.001
3+	67 552 (38.7)	20895 (37.5)	33263 (39.1)	13394 (39.7)	
Primary diagnosis category					
Other	123 290 (70.7)	38897 (69.8)	60880 (71.6)	23513 (69.7)	<.001
Appendicitis	611 (0.4)	180 (0.3)	286 (0.3)	145 (0.4)	
Asthma	5613 (3.2)	2054 (3.7)	2301 (2.7)	1258 (3.7)	
CNS shunt	1213 (0.7)	332 (0.6)	606 (0.7)	275 (0.8)	
Dehydration/GI infection	4730 (2.7)	1593 (2.9)	2183 (2.6)	954 (2.8)	
Fever	1543 (0.9)	569 (1)	761 (0.9)	213 (0.6)	
Resp, Lower (Bronchiolitis)	5823 (3.3)	2051 (3.7)	2395 (2.8)	1377 (4.1)	
Resp, Lower (Pneumonia)	13 149 (7.5)	3802 (6.8)	6513 (7.7)	2834 (8.4)	
Resp, Upper	2288 (1.3)	811 (1.5)	1014 (1.2)	463 (1.4)	
Seizure	10 939 (6.3)	3842 (6.9)	5311 (6.2)	1786 (5.3)	

© 2025 Carroll AR et al. *JAMA Network Open*

Sickle cell	5214 (3)	1560 (2.8)	2746 (3.2)	908 (2.7)	
Prior select procedure in the 6 mo before admission					
0	88 797 (50.9)	30107 (54.1)	41796 (49.2)	16894 (50.1)	<.001
1+	85 616 (49.1)	25584 (45.9)	43200 (50.8)	16832 (49.9)	
Prior utilization in 6 mo before admission					
1	58 298 (33.4)	19551 (35.1)	28252 (33.2)	10495 (31.1)	
2	38 145 (21.9)	12385 (22.2)	18551 (21.8)	7209 (21.4)	
3	24 335 (14)	7928 (14.2)	11787 (13.9)	4620 (13.7)	
4+	53 635 (30.8)	15827 (28.4)	26406 (31.1)	11402 (33.8)	
Service category					
Medical	149 024 (85.4)	47 131 (84.6)	73055 (86)	28838 (85.5)	<.001
Surgical	25 389 (14.6)	8560 (15.4)	11941 (14)	4888 (14.5)	

[a]RAM = Recent Admission Model
[b]AUROC = area under the receive operator curve

© 2025 Carroll AR et al. *JAMA Network Open*
