# Supplementary material for: Validation of 30-Day Pediatric Hospital Readmission Risk Prediction Models
Source: JAMA Netw Open. 2025 Feb 13;8(2):e2459684. doi: 10.1001/jamanetworkopen.2024.59684 (PMC11826366; doi:10.1001/jamanetworkopen.2024.59684)
Supplement: Supplement 2. — Data Sharing Statement [file jamanetwopen-e2459684-s002.pdf]

Data Sharing Statement

Carroll. Validation of 30-Day Pediatric Hospital Readmission Risk Prediction Models. *JAMA Netw Open*. Published February 13, 2025. doi:10.1001/jamanetworkopen.2024.59684

Data
Data available: No
